# Tibial shaft fractures: A bibliometric analysis of the top 50 most cited publications

**DOI:** 10.1016/j.jor.2025.06.020

**Published:** 2025-06-30

**Authors:** Ethan Gilkinson, Ben Murphy, Niall McGoldrick, John Quinlan

**Affiliations:** Department of Trauma & Orthopaedic Surgery, Tallaght University Hospital, Tallaght, Dublin 24, Ireland

**Keywords:** Tibial shaft fractures, Bibliometric analysis, Treatment outcomes

## Abstract

**Background:**

Tibial shaft fractures (TSFs) are a frequent presentation within orthopaedics, most often occurring in young males following a high energy trauma. The appropriate management of these fractures is crucial, ensuring patients have the best chance at achieving a functional recovery. In spite of the vast number of studies conducted on TSFs, the quality of scientific evidence and various trends related to this injury remains poor. The aim of our analysis is to identify these trends within the 50 most cited publications pertaining to TSFs.

**Methods:**

A bibliometric analysis was carried out using the Web of Science platform to report the 50 most cited publications associated with TSFs. The publications were screened using definite inclusion and exclusion criteria. Features such as institution, authorship, level of evidence and patient demographics were reported.

**Results:**

The 50 most cited publications in total listed 10, 407 citations, with the most cited study receiving 1075 citations. Treatment outcomes (66 %) and surgical technique (32 %) were the primary focus of the majority of the publications. Retrospective cohort and case control studies made up almost half of the studies (48 %), many being of level III evidence. The McMaster University Hospital was the institution that produced the greatest number of studies, with the Journal of Bone and Joint Surgery having published most of the articles.

**Conclusion:**

Many publications exist related to TSFs, primarily focusing on treatment outcomes and how to minimize the incidence of non-union following surgical fixation. However, a significant number of the publications were of low evidence and were identified as being retrospective in nature. Following this analysis, future research should aim to produce studies of a higher quality and focus on areas that carry the most promise or influence, such as the use of recombinant human bone morphogenetic protein-2 in intramedullary nailing of TSFs.

## 1 Introduction

Tibial shaft fractures (TSFs) are the most commonly fractured long bone with an incidence of 16.9/100,000/year. More frequent in males, the incidence of this injury occurs most often between the ages of 10 and 20. In comparison, females are found to have a higher frequency between the ages of 30 and 40.^1^ A bimodal pattern is often witnessed with TSFs, involving mechanisms of either low or high energy. Low energy injuries often result in spiral fractures of the tibial shaft, caused by a torsional force with minimal soft tissue damage. High energy injuries secondary to direct trauma more commonly cause transverse or comminuted fractures with significant soft tissue injury.^2^^,^^3^ With TSFs largely occurring in those of working age, this results in a substantial financial impact on the patient, due to a loss of productivity and medical expenses.^4^ They can be associated with both short and long term complications, spanning from acute compartment syndrome to chronic knee pain.^5^

Given the morbidity associated with these fractures, it is imperative that patients who have suffered a TSF have are promptly diagnosed and treated accordingly. Non-surgical options may involve closed reduction and immobilization in a cast. Surgical treatment may include external fixation, intra-medullary nailing (IMN) or various plating techniques, depending on the personality of the fracture. Although non-operative measures avoid the risks associated with surgery, surgical fixation offers a number of benefits such as reduced pain, quicker time to union, decreased risk of non-union and a faster recovery enabling patients to return to employment and their pre-injury functional level sooner.^6^

A bibliometric analysis (BA) aims to review academic productivity via scientific publications. It is focused on the quantitative analysis of citations, the implication of a particular area of research and the geographical location from where it was published.^7^ It is often used within the medical field to highlight new trends, identify collaboration patterns and inspect the academic structure of certain areas using existing literature.^8^ This intends to provide scholars with a comprehensive summary of a particular topic, allow the identification of gaps in research and encourage the development of new ideas for areas of investigation that will contribute to the field.^9^

The aim of our study was to assess current research trends and review the features of the 50 most cited publications relating to the epidemiology and management of TSFs. The authors of this paper anticipated a sharp rise in the number of publications associated with TSF's in recent years given the development of new fracture fixation techniques. We also predicted that the majority of the studies would be retrospective in nature and would consist of papers mainly of level of evidence III and lower.

## 2 Methods

Using the Web of Science platform (Clarivate Plc, Boston, MA, USA), a literature search was carried out in May 2025 using the terms “Tibial Shaft Fracture” or “Tibia Shaft Fracture” or “Diaphyseal Tibial Fracture” or “Diaphyseal Tibia Fracture”. The results were arranged in order of most citations prior to a preliminary assessment by the primary author. Each publication was inspected based on their title and abstract, with the application of a definite inclusion and exclusion criteria.

The inclusion criteria applied involved publications that were in the English language and had their full text accessible for review. The exclusion criteria employed eliminated review articles, cadaveric/anatomical/animal studies and publications involving proximal, distal and peri-prosthetic tibial fractures.

The top 50 most cited publications pertaining to TSFs were exported to Microsoft Excel (Microsoft Corporation, Redmond, WA) before a more extensive review was carried out by the primary and second author. A level of evidence (LOE) was assigned to each publication using a criteria designed by Wright et al.^10^ When reviewing each publication, the following features were recorded – authors, institution, number of participants, LOE, participant demographics, alongside each of the studies’ primary aim related to TSFs.

## 3 Results

**Fig. 1:**
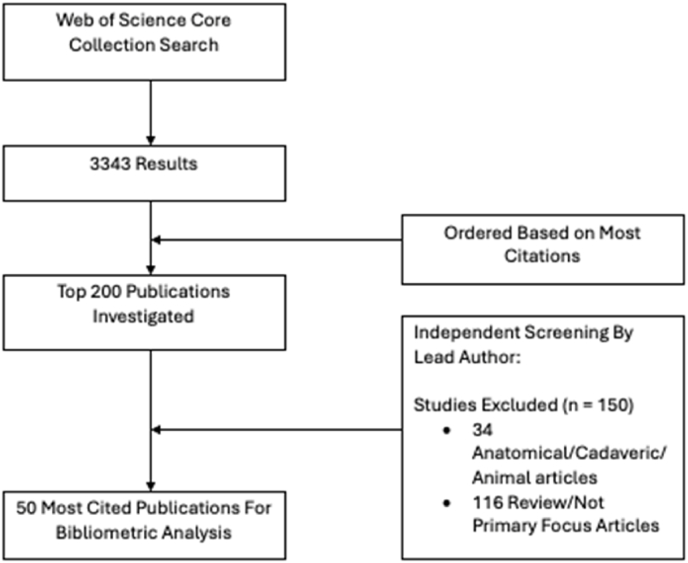
Flowchart demonstrating screening process for selected articles.

A total of 3343 publications were noted on the initial literature search relating to TSFs. These studies were arranged in order of most to least citations, then the top 200 publications were examined by two independent authors (EG, BM). Following the application of the inclusion/exclusion criteria, 150 publications were removed and 50 were selected for further investigation (Fig. 1).

**Fig. 2:**
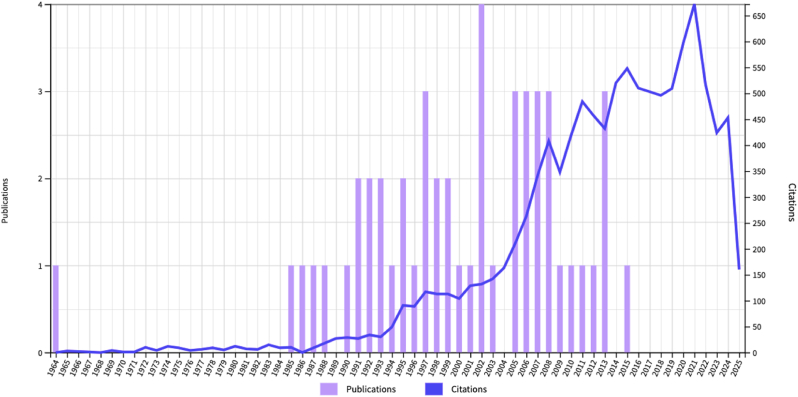
Publications/Citations relating to TSFs. **(PLEASE USE COLOUR)**.

The total number of citations was 10,407 amongst the included studies. The most cited publication was cited 1075 times and the least-cited publication was cited 119 times (Fig. 2). Among the top 50 most-cited publications pertaining to TSFs, treatment outcomes was the most common primary focus (n = 33; 66 %). Other notable study areas for the remaining publications included surgical technique (n = 16; 32 %) and epidemiology (n = 7; 14 %) related to TSFs.

Almost half of the included studies were noted to be retrospective in nature (n = 24; 48 %). 18 (36 %) publications were retrospective cohort studies and 6 (12 %) were retrospective case control studies. The remaining publications mainly consisted of prospective studies (n = 15, 27 %), consisting of randomised cohort and observational studies. Using Wright et al.’s scoring system to assign a scientific level of evidence (LOE) for each publication, over half of the studies were of level 3 evidence (n = 29; 58 %) and 26 % (n = 13) were of level 2 evidence.

**Fig. 3:**
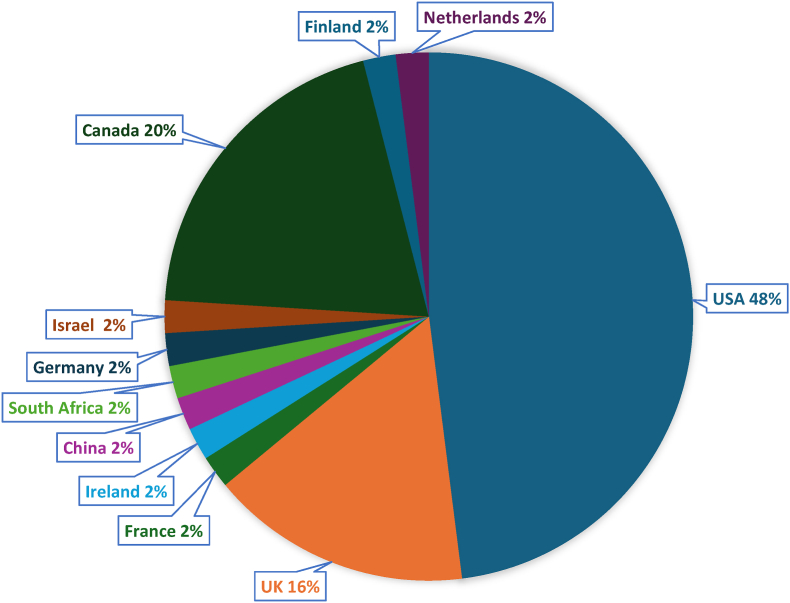
Publications by country of origin. **(PLEASE USE COLOUR)**.

A total of 12, 321 patients were recorded in the top 50 cited publications relating to TSFs. Patient demographics were reported in 38 publications out of 50, with most being published in the United States (Fig. 3). The majority of participants were of male gender (n = 8354, 67.8 %). The mean age of the patients involved in all studies was 35.46 years. Among the top 50 most cited publications, the institution with the most publications was the McMaster University Hospital in Canada (n = 5). The institution which published the article with the most citations was The University KwaZulu Natal, located in South Africa (n = 1075) (Fig. 3). The authors with the most publications were M Bhandari (n = 7) and M Swiontkowski (n = 6) respectively (Table 1).

**Table 1:** The top 5 publishing authors for the most cited publications associated with TSFs.

AUTHOR	PUBLICATIONS	% of 50
Bhandari, M	7	14
Swiontkowski, M	6	12
Schemitsch, E	5	10
Guyatt, G	5	10
Keating, J	3	6

When reviewing the most recently published literature, the main objective of these papers was predominantly focused on the epidemiology and management of non-union in TSFs.

The time frame in which most papers were published was between 2002 and 2008 (n = 17), with the most publications in a single year being in 2002 (n = 4). The top 3 most-cited articles were in 1994, 2002 and 2005. The Journal of Bone & Joint Surgery (Impact Factor 5.3) had the highest number of publications, with 23 articles. This was then followed by Clinical Orthopaedics and Related Research (Impact Factor 4.4) and the Journal of Orthopaedic Trauma (Impact Factor 1.6) (Table 2).

**Table 2:** The top 5 journals with highly-cited publications related to TSFs.

JOURNAL	IMPACT FACTOR	PUBLICATIONS
Journal of Bone and Joint Surgery	5.3	23
Clinical Orthopaedics and Related Research	4.4	7
Journal of Orthopaedic Trauma	1.6	7
Injury International Journal of the Care of the Injures	2.2	3
BMC Musculoskeletal Disorders Journal	2.2	2

## 4 Discussion

Our bibliographic analysis demonstrates a rise in the number of articles related to TSFs which have been published in recent years. It highlighted that the majority of articles with the highest number of citations were focused primarily on treatment outcomes but still were of a relatively low level of evidence i.e. level III and lower.

Our study's analysis confirms that the aetiology of TSFs is most commonly due to high energy trauma. More frequently occurring in young males, these fractures are most often caused by vehicle collisions, sports injuries or falls from a height.^11^ The literature most cited within this study commonly focused on open fractures, injuries that occur as a result of high energy impact. However, we also know that TSFs can be bimodal in terms of energy expended to cause the fracture, also occurring in elderly females due to simple falls.^12^ With an increased life expectancy in a globally aging population, this cohort possess a more active lifestyle than previous generations. The authors of this paper would hypothesise that further work will be published in this area, primarily focusing on the aetiology and management of low energy injuries in the geriatric population. We also hypothesise that the rate of open fractures should see a gradual decline secondary to the introduction of road safety campaigns and preventative measures, reducing the volume of road traffic accidents.^13^

The complications of TSFs are varied, ranging from neurovascular compromise, compartment syndrome, non-union, malunion and osteomyelitis. Therefore, the management of these fractures is vital for orthopaedic surgeons.^6^ Intramedullary nailing (IMN) was the main form of surgical management discussed throughout the 50 publications, investigating numerous techniques and post-operative outcomes. However, the main topic of debate was whether reamed IMN was more successful in TSFs. A number of randomized controlled trials (RCT) have been conducted comparing the effects of reamed and un-reamed IMN. A meta-analysis has been carried out on these trials, reporting a large decrease in the risk of non-union in reamed IMN of TSFs.^14^ We noted only a single RCT on IMN amongst the top 50 cited publications, suggesting citation number does not always represent studies of the highest quality.

Treatment outcomes was the most discussed primary aim of all the studies in this analysis (66 %). The majority of these publications focused on the development of fracture non-unions following surgery, with most requiring secondary intervention. They commonly discussed predictors for tibial shaft non-union. Some confirmed already known risks such as the presence of a fracture gap post fixation. Others aimed to highlight lesser known predictors they believe to be significant, such as cortical continuity.^15^ 6 % of the 50 publications discussed the benefits of using recombinant human bone morphogenetic protein-2 (rhBMP-2) alongside IMN in open tibial shaft fractures. Of note, a paper on this topic obtained the highest number of citations out of the 50 publications (n = 1075), suggesting its strong influence on the operative management of TSFs. Further analysis uncovered studies which have already shown that the use of rhBMP-2 accelerates fracture healing and reduces the need of any secondary intervention by avoiding non-union.^16^ A recent study by Choi et al. reported that the combination of autogenous bone graft with rhBMP-2 had excellent radiological and functional outcomes in patients with long bone non-union, with no adverse effects. This was a small prospective series but highlights a promising area for further investigation.^17^

The majority of the top 50 most cited publications related to TSFs were of level III evidence. They were also noted to be retrospective in nature, consisting of randomised cohort or case studies. This result is comparable to the current orthopaedic research within various fields, which also possess a low level of evidence.^18^ There were only 2 publications deemed to be of level I evidence throughout this analysis. Both studies took place at the McMaster University Hospital, one being a systematic review and the other a RCT, both focusing on the benefits of reamed vs un-reamed IMN in TSFs. According to Cunningham et al., the level of evidence within orthopaedics has improved in recent years. In highlighting the low level of evidence seen in TSF publications with our analysis, we would hope to drive more meaningful research in this area – with the ultimate aim of improving patient care.^19^

The Journal of Bone and Joint Surgery was noted in our study as having the greatest number of publications related to TSFs. Well-renowned for its contribution to research, especially within the area of orthopaedic trauma, this journal has been assigned a particularly high impact factor which is known to be directly linked to the journals citation count.^20^ Citation frequency can be influenced by a number of factors such as the time since it was published, dissemination bias and institutional prestige. However, it is still employed to serve as an objective measure to classify literature and avoid ambiguity within research.^21^

## 5 Strengths & limitations

This study is not without limitations. Publications with a high number of citations are not always a true representation of a paper's quality. We demonstrated this by identifying that the most cited literature related to TSFs consisted mainly of studies of a low quality (level III) as per Wright et al. By solely focusing on citation count, our analysis runs the risk of neglecting publications that possess a higher LOE with greater potential of advancing surgical practice. The prospect that some literature may have been overlooked using the Web of Science platform also exists. Publication bias may be applicable following the exclusion of articles that are not published in the English language.

The strengths of our study lie within the identification of various research gaps and highlighting emerging trends in the research field of TSFs. This includes the promotion of higher quality research, particularly within areas that show promise, such as the use of rhBMP-2 in IMN. By doing so this will ultimately drive innovation, enhance patient results and improve of overall efficacy of the healthcare system. This analysis also revealed the most influential institutions, journals and authors pertaining to TSFs. Through this, practical research networks can be established, fostering interdisciplinary collaborations to bring about the most optimal results.

## 6 Conclusion

Research into the epidemiology and treatment of TSFs continues to be a popular topic within the field of orthopaedics. We have identified the journals, countries and authors that have made the greatest contribution to research within this area. The use of highly cited articles related to these fractures yielded studies of low quality, despite being published in journals that possess a high impact factor. Whilst citation frequency does not always dictate scientific quality, it does identify the literature which has made the largest impact to the area of interest. The material discussed throughout our analysis will prove useful in promoting future research and advancing the surgical management of TSFs.

## CRediT authorship contribution statement

**Ethan Gilkinson:** Conceptualization, Formal analysis, manuscript composition. **Ben Murphy:** Formal analysis, manuscript composition. **Niall McGoldrick:** Manuscript review. **John Quinlan:** Manuscript review, senior author.

## Guardian/patient's consent

Not applicable.

## Ethics

1) This material is the authors' own original work, which has not been previously published elsewhere.
2) The paper is not currently being considered for publication elsewhere.
3) The paper reflects the authors' own research and analysis in a truthful and complete manner.
4) The paper properly credits the meaningful contributions of co-authors and co-researchers.
5) The results are appropriately placed in the context of prior and existing research.
6) All sources used are properly disclosed (correct citation). Literally copying of text must be indicated as such by using quotation marks and giving proper reference.
7) All authors have been personally and actively involved in substantial work leading to the paper, and will take public responsibility for its content.

## Funding

This research did not receive any specific grant from funding agencies in the public, commercial, or not-for profit sectors.
